# Retraction Note: NR1B2 suppress kidney renal clear cell carcinoma (KIRC) progression by regulation of LATS 1/2-YAP signaling

**DOI:** 10.1186/s13046-026-03839-8

**Published:** 2026-10-07

**Authors:** Lei Yin, Wenjia Li, Guangchun Wang, Heng Shi, Keyi Wang, Huan Yang, Bo Peng

**Affiliations:** 1 Department of Urology, School of Medicine, Shanghai Tenth People’s Hospital, Tongji University, Shanghai, China; 2 Shanghai Institute of Cardiovascular Disease, Zhongshan Hospital, Fudan University, Shanghai, China; 3 Department of Urology, Shanghai Tenth People’s Hospital, Nanjing Medical University, Nanjing, China; 4 Department of Urology, Tongji Medical College, Tongji Hospital, Huazhong University of Science and Technology, Wuhan, China

**Retraction Note: J Exp Clin Cancer Res 38, 343 (2019)**

**https://doi.org/10.1186/s13046-019-1344-3**

The Editor-in-Chief has retracted this article. Concerns were raised regarding a number of figures, specifically:

- Figure 3I: the upper left section of PLKO/Migration panel and the lower right section of PLKO/Invasion panel appear to overlap;
- Figure 5B: the 786-O: SH-NR1B2 band of NR1B2 and the SW839: OE-NR1B2 band of P-YAP appear to overlap when flipped;
- Figures 3H and 5G: the OE-NR1B2/Invasion and PWPI/Migration panels of Fig. 3H appears to overlap with the OE-NR1B2(+) Si-YAP(-)/Invasion and OE-NR1B2(-) Si-YAP(-)/Migration panels of Fig. 5G, respectively.

The authors provided some source data on request from the Publisher, but this was insufficient to address the concerns raised. The Editor-in-Chief therefore no longer has confidence in the reliability of the data reported in the article.

None of the authors have responded to any correspondence from the Publisher regarding this retraction.

